# Dystrophin Cardiomyopathies: Clinical Management, Molecular Pathogenesis and Evolution towards Precision Medicine

**DOI:** 10.3390/jcm7090291

**Published:** 2018-09-19

**Authors:** Domenico D’Amario, Aoife Gowran, Francesco Canonico, Elisa Castiglioni, Davide Rovina, Rosaria Santoro, Pietro Spinelli, Rachele Adorisio, Antonio Amodeo, Gianluca Lorenzo Perrucci, Josip A. Borovac, Giulio Pompilio, Filippo Crea

**Affiliations:** 1 Dipartimento di Scienze Cardiovascolari e Toraciche, Fondazione Policlinico Universitario A. Gemelli IRCCS, Università Cattolica del Sacro Cuore, Largo A. Gemelli 8, 00100 Rome, Italy; fra.canonico@virgilio.it (F.C.); filippo.crea@unicatt.it (F.C.); 2 Centro Cardiologico Monzino-IRCCS, Unit of Vascular Biology and Regenerative Medicine, Via Carlo Parea 4, 20138 Milan, Italy; agowran@ccfm.it (A.G.); elisa.castiglioni@cardiologicomonzino.it (E.C.); davide.rovina@cardiologicomonzino.it (D.R.); rosaria.santoro@cardiologicomonzino.it (R.S.); pietro.spinelli.bio@gmail.com (P.S.); gianluca.perrucci@cardiologicomonzino.it (G.L.P.); Giulio.Pompilio@cardiologicomonzino.it (G.P.); 3 Department of Paediatric Cardiology and Cardiac Surgery, Bambino Gesù Hospital (OPBG), Piazza S. Onofrio 4, 00165 Rome, Italy; rachele.adorisio@opbg.net (R.A.); antonio.amodeo@opbg.net (A.A.); 4 Department of Clinical Sciences and Community Health (DISCCO), University of Milan, Via Francesco Sforza 35, 20122 Milan, Italy; 5 Department of Pathophysiology, University of Split School of Medicine, Soltanska 2, 21000 Split, Croatia; jborovac@mefst.hr

**Keywords:** cardiomyopathy, dilated, cardiomyopathy in muscular dystrophy, Duchenne muscular dystrophy, dystrophin, heart failure, personalized medicine

## Abstract

Duchenne’s muscular dystrophy is an X-linked neuromuscular disease that manifests as muscle atrophy and cardiomyopathy in young boys. However, a considerable percentage of carrier females are often diagnosed with cardiomyopathy at an advanced stage. Existing therapy is not disease-specific and has limited effect, thus many patients and symptomatic carrier females prematurely die due to heart failure. Early detection is one of the major challenges that muscular dystrophy patients, carrier females, family members and, research and medical teams face in the complex course of dystrophic cardiomyopathy management. Despite the widespread adoption of advanced imaging modalities such as cardiac magnetic resonance, there is much scope for refining the diagnosis and treatment of dystrophic cardiomyopathy. This comprehensive review will focus on the pertinent clinical aspects of cardiac disease in muscular dystrophy while also providing a detailed consideration of the known and developing concepts in the pathophysiology of muscular dystrophy and forthcoming therapeutic options.

## 1. Introduction

Traditionally muscular dystrophies (MD) were predominantly considered as skeletal muscle pathologies. However, now it is accepted that MD also involve important pathological alterations in cardiac functions [1]. In Duchenne MD (DMD), cardiac complications have recently escalated, since the application of improved therapies succeeded in prolonging MD patients’ lifespan. Indeed, a substantial fraction of patients with MD develop cardiomyopathy (CM) that limits survival [2]. DMD and Becker MD (BMD) are characterized by the total or partial loss of dystrophin protein expression, respectively. The varying degree of dystrophin expression explains the stark contrast of the respective clinical courses of DMD and BMD; severe with early onset in the former and latter presenting later in life and often entails less severe symptoms. Dystrophin deficit in myocytes cause a general disorganization of the dystrophin-associated protein complex (DAPC) that normally anchors the cortical actin cytoskeleton and the plasma membrane (PM) to the extracellular matrix (ECM). MD patients’ skeletal muscle cells are seemingly more susceptible to membrane damage caused by physiological contractions of myocytes since most patients develop skeletal myopathy before cardiomyopathy. Although with the continual evolution of advanced imaging modalities this assertion is under question. Currently, no predictive measures for muscular dystrophy cardiomyopathy (MD-CM) occurrence, rate-of-progression, or clear mechanistic insights exist. In the myocardium, absent or defective dystrophin protein expression is associated with myocarditis, fibro-adipose tissue replacement, cardiomyocyte death, and loss of function [3]. There is considerable heterogeneity in MD disease. DMD is the most common early-onset severe form of MD while the less frequent BMD is a relatively milder disease phenotype with a later onset. The degree of disruption in the dystrophin gene-reading frame is one explanation for this difference; however, this rule is often not applicable [4]. In the majority of MD patients, progressive skeletal muscle wasting is also accompanied by the development of cardiac abnormalities, such as inflammatory cell activation, cardiomyocyte death, fibro-fatty infiltration, wall motion malfunctions, left-ventricular dysfunction, and arrhythmias; leading to dilated cardiomyopathy and heart failure (HF) [5]. MD-CM phenotypes are also clinically divergent, including the age of onset, progression rate, and severity [1,6,7]. Numerous studies have failed to determine distinct genotype-phenotype correlations or an underlying mechanism to explain these heterogeneous cardiac phenotypes. Furthermore, the presence of cardiomyopathy in 10–60% of carrier females (CFs), females carrying dystrophin gene mutations, known as symptomatic CFs [8], lends further weight to the necessity for changes in research approach. A significant limiting factor in the development of therapeutic strategies for MD-CM is the lack of suitable human-tissue-based models capable of recapitulating disease phenotypes and providing insights on mechanisms linked to MD-CM onset and progression. Indeed, seemingly promising findings uncovered in numerous mouse models have failed to translate to humans, leaving much scope for the collective action of research scientists, medical teams, MD patients, CFs, family members and friends. The present review assembles and collates information obtained from clinical and pre-clinical research on MD-CM and deliberates the incorporation of elements from the precision medicine field to future clinical therapeutic strategies.

## 2. Clinical Aspects

**Figure 1:**
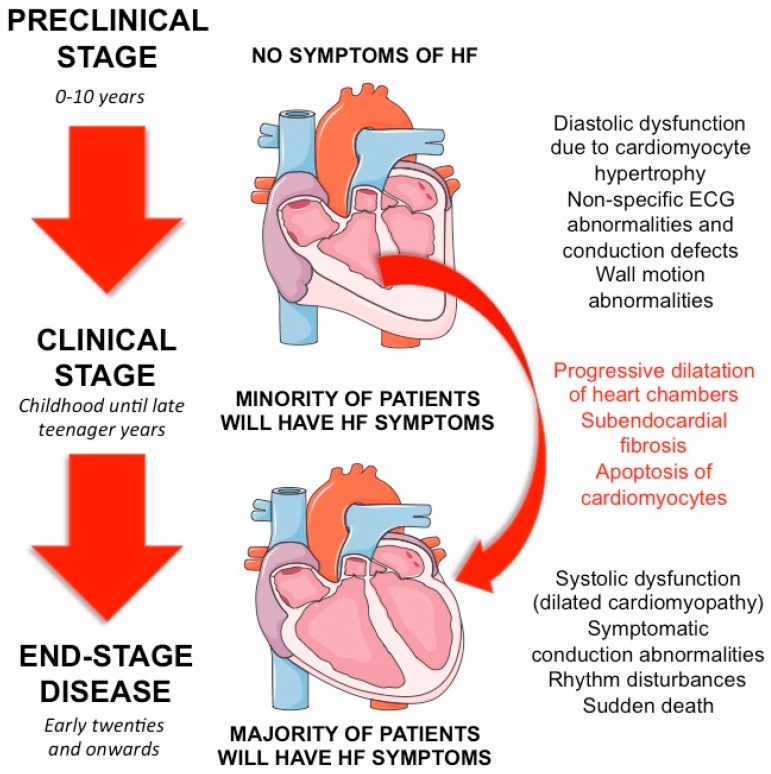
Natural course of cardiovascular impairment in patients with Duchenne’s muscular dystrophy divided in three cardinal phases: preclinical phase of disease when patients have no symptoms of heart failure (HF) and cardiac investigations reveal non-specific findings; transitional phase of the disease leading to symptoms and detectable signs of cardiac impairment; the final phase involving clear clinical manifestations of disease and significant cardiac impairment leading to end-stage cardiomyopathy and death.

At the onset, affected children usually present with waddling gait, lordotic posture, calf hypertrophy, and positive Gower’s sign (difficulty in rising from the floor, spreading their legs, and using their hands to climb up their thighs to help them to an upright position). Often dystrophic toddlers are late to walk, and the calf muscle can show signs of hypertrophy. Motor performance reaches a plateau between three and six years; deterioration begins at six to eight years. Self-propulsion and the ability to maintain posture is possible for some time, however lordosis and scoliosis become evident usually by eight years of age. Between nine and twelve years of age, the majority of DMD patients are wheelchair-bound: yet upper limb function can be preserved for long periods after loss of ambulation. Decreased pulmonary function, as a result of respiratory muscle weakness and chest deformity (kyphoscoliosis), along with respiratory infections, predisposes MD patients to respiratory failure. Respiratory issues are considered the most common limiting factor to put forward these patients as candidates for heart transplantation and are the leading cause of death in MD patients. However, cardiac complications e.g., predominantly HF and arrhythmias, are steadily increasing and nowadays are on par with respiratory failure as a major cause of death, thus impelling the definition of new therapeutic strategies [9]. Clinical manifestations of HF (fatigue, weight loss, vomiting, abdominal pain, sleep disturbance, and inability to tolerate daily activities) are often underrecognized owing to musculo-skeletal limitations. This aspect is reported to be particularly evident in CF [10]. Connuk et al. showed that although DMD and BMD patients have similar ages at the onset of CM (14.4 ± 2.3 vs. 14.6 ± 2.0 years) and have a comparable rate of cardiomyopathy at diagnosis (30% vs. 33%), the DMD group had a significantly higher mortality rate than BMD patients. This difference in survival may be attributable to the level of physical activity which is higher in patients with BMD; respiratory muscle function which is better in patients with BMD; although controversial, BMD patients are considered for heart transplantation in certain treatment centres; lastly more rapid heart degeneration due to the lack of dystrophin in DMD compared to reduced levels in BMD [11,12]. Although guidelines on the cardiac management of DMD patients are available, and despite a broader awareness of the importance of MD-CM, standards of care remain highly variable and controversially dependent on arbitrary factors [1]. Based on the Cooperative International Neuromuscular Research Group (CINRG) registry, that collected natural history data from participants to the CINRG-Duchenne Natural History Study (DNHS), 32% of patients did not undergo echocardiography by the time of their first diagnosis while CM was present in 34% of patients who had an echocardiogram at baseline. Moreover, half of the patients with CM did not receive any specific treatment at the moment of diagnosis [13]. A natural course of disease in respect to symptoms and underlying cardiac pathophysiology is presented in Figure 1.

## 3. Dystrophin—From Gene to Protein

### 3.1. The Dystrophin Gene and Its Transcript

The Duchenne muscular dystrophy gene (*DMD*, italics differentiate it from Duchenne’s MD) is the largest gene present in the human genome. Gene linkage and positional cloning mapped *DMD* to the short arm of the X-chromosome (Xp21) [14,15]. *DMD* spans approximately 2.5 megabases that correspond to 1.5% of the X chromosome and approximately 0.1% of the entire human genome [4,16,17,18]. A majority of the gene sequence (99%) consists of introns, while the coding sequence is divided into 79 constitutive exons. The full-length messenger RNA is 14,000 base pairs (bp) long and is transcribed from three different promoters: the upstream brain (B), muscular (M) and purkinje (P) promoters drive the transcription of three full-length isoforms (Dp427) that have the same number of exons but are expressed in a tissue-specific manner [4] reflected in the name assigned to each promoter that refers to the principal tissue expression site. In particular, the B promoter guides the expression of dystrophin mainly in hippocampal and cortical neurons [19,20] while the P promoter is active in cerebellar Purkinje cells [21,22]. Lastly, the M promoter is utilized to express dystrophin in skeletal and cardiac myocytes [23] and at very low levels in glial cells [24]. In addition to these full-length mRNAs, *DMD* gives rise to another four different transcripts each starting from a specific promoter. These promoters are located in the gene body; within intron 29 (R, retinal isoform, Dp260), intron 44 (B3, brain-specific isoform, Dp140), intron 55 (S, Schwann cell isoform, Dp116), and intron 62 (G, general isoform, Dp71) [4,25]. *DMD* has homology with other different gene classes. In particular, the whole coding sequence shows high similarity with the utrophin gene [26]: the 5′ end and central parts have homology with proteins of the spectrin family (e.g., α-actinin) while the 3′ end is homologous to dystrobrevin (a post-synaptic protein) [25]. In addition to these isoforms, alternative splicing of *DMD* produces other different isoforms that are tissue-specific, increases the diversity of the dystrophin protein, and explains the complexity of expression regulation for tissue-specific functions.

### 3.2. The Dystrophin Protein

The full-length dystrophin protein (that arises from the B, M, or P promoters) is a large rod-shaped protein containing about 3685 amino acids with a molecular weight of 427kDa. This large protein folds into four different protein domains: the amino-terminal domain, the central rod-like domain, the cysteine-rich domain, and the C-terminal domain [4,25]. The amino-terminal region, encoded by exons 1–8, shows high homology with a family of actin-binding proteins, in particular, α-actinin and β-spectrin [27]. This domain is considered the principal region of interaction with the actin cytoskeleton since three actin-binding sites have been found in this section. However, other downstream sites have been identified that impact dystrophin-actin interactions [28]. The rod-like domain is the largest part of the protein and is encoded by exons 9–63 [29]. It consists of 24 units with high homology to the repeated regions in the α-spectrin protein and is predicted to form triple helical coiled coils [30]. Four short proline-rich spacers, the so-called hinge domains that provide elasticity to the protein [31], interrupt this region. The rod domain also contains a second actin-binding motif [32] and interacts with anionic lipids [33], neuronal NOS (nNOS) [34,35], and polarity regulating kinase (PAR-1b). The cysteine-rich domain is encoded by exons 64–69 and contains two EF-hand-like modules that are bound by WW and ZZ domains [36,37] which are important for protein–protein interaction and for the stabilization of dystroglycan binding [38]. The C-terminal region, encoded by exons 70–79 [39], is fundamental for the binding with a group of extracellular (α-dystroglycan), integral membrane (sarcoglycans and β-dystroglycans), and cytoplasmic (syntrophins and dystrobrevins) proteins to create the DAPC [40,41]. Full-length dystrophin is associated with the sarcolemma of skeletal and cardiac myocytes and interacts with the actin cytoskeleton and the dystrophin-associated proteins present in the DAPC, creating a bridge between the extracellular matrix and the cytoskeleton. All the shorter dystrophin proteins lack the N-terminal actin-binding domain but retain a portion of the rod domain (with the exception of Dp71), the cysteine-rich domain and the C-terminal domain that has the binding site for dystroglycan, dystrobrevin, and syntrophin. The expression and function of the shorter dystrophin isoforms seem to be tissue-specific. Dp260 is highly present in the retina, and its lack is associated with electroretinogram anomalies [42,43,44]. Dp140 is expressed in the brain, retina, and kidneys where it appears to be involved in brain development and blood flow regulation [45,46,47]. Dp116 is produced in Schwann cells, however, its role in this context is poorly understood [48]. Dp71 is ubiquitously expressed, it is found in most non-muscle tissues such as brain, kidney, liver, retina, and lung. Significantly, it is also produced in cardiac but not in skeletal myocytes. This isoform shows multiple roles in different cellular processes, including cell adhesion, water homeostasis, nuclear architecture and cell division. The lack of this protein has been associated with mental retardation and retinal dysfunctions [49].

### 3.3. DMD Mutations

Since *DMD* is the largest gene of the human genome, the mutation rate is relatively high and one-third of MD cases are the consequence of de novo mutations [50]. The large intron size has been suggested as one of the principal causes of the high mutation rate [4,25]. The most common alteration of *DMD* are intragenic deletions spanning one or more exons and accounting for approximately 65% of *DMD* mutations. Duplication of one or multiple exons accounts for 10% of all *DMD* mutations. Both deletions and duplications can arise anywhere in the gene, however they tend to cluster in two regions. The principal hotspot is between exons 45 and 55, with the breakpoint frequently lying in intron 44, while the second hotspot spans from exons 3 to 19, with the breakpoint in intron 2 or 7. These three introns are highly conserved and they may contain regulatory sequences. Small mutations including, point mutations (nonsense and missense), indels and rare types (e.g., small inversions or complex small rearrangements) account for a quarter of *DMD* mutations. While deep intronic alterations and complex rearrangements account for no more than 1%. It is difficult to establish the functional consequences of all the mutations based only on each specific alteration or position of the mutation in the *DMD* gene. Indeed, there is no simple relationship between the size of the deletion/duplication, the region or domain, and the subsequent clinical phenotype. The functional consequences of the mutation appear to be mainly associated with the maintenance of the correct open reading frame that allows the accurate and full translation of the mRNA product of the *DMD* gene. Based on these observations, Monaco et al. [51] proposed the so-called ‘reading frame rule’. Mutations associated with *DMD* disrupt the reading frame directly (nonsense) or indirectly (all other types) causing premature stop codons and, therefore, an early-truncated and non-functional protein product. On the other hand, *DMD* mutations that preserve the reading frame (in-frame) allow the production of an abnormal but partially functional dystrophin protein and are associated with BMD. Although shorter or longer in the central domain, this truncated and partially functional protein maintains the N- and C-terminal regions that are fundamental for the connection of actin with the extracellular matrix. Conversely, frame-shift mutations result in unstable mRNA that cause the production of low levels of truncated proteins. The reading frame hypothesis holds for more than the 90% of MD patients and is normally used as a diagnostic confirmation of dystrophinopathies and for the differential diagnosis of DMD and BMD. However, exceptions to this rule do exist and include both BMD patients with out-of-frame deletions/duplications or cases of DMD with in-frame mutations. Namely, for BMD individuals with out-of-frame *DMD* mutations, the reading frame could be restored through alternative splicing events (e.g., exon skipping) that permit the production of a truncated form of dystrophin. Deletions in the 5′ region of *DMD* are also associated with DMD if they are in-frame; seemingly due to an altered actin-binding site that prevents dystrophin–actin cytoskeleton interaction.

## 4. Pathophysiology—Alternatives to the Structural Hypothesis

**Figure 2:**
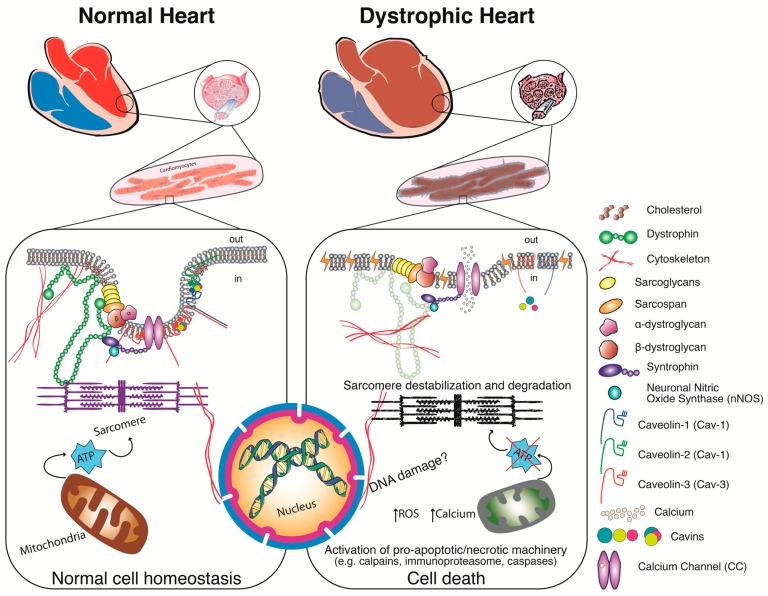
MD-CM pathological signalling mechanisms. A schematic depicting the pathological signalling resulting from mutations in the dystrophin gene. Muscular dystrophy mutations cause dystrophin protein deficiency ranging from no expression to the generation of semi-functional truncated isoforms which result in clinically severe (e.g., Duchenne) or milder (e.g., Becker) forms of muscular dystrophies respectively. Downstream effects of altered dystrophin protein are: over activation of ion channels and rises in intracellular ion concentrations e.g., calcium; fragile sarcolemmal membranes which are subject to rupture due to sustained myocyte contraction resulting in sarcolemal micro-ruptures (yellow strikes) through which cytosolic components leak out. Further downstream signalling can be instigated such as: disturbed sarcolemmal lipid and protein content/arrangement, mitochondrial dysfunction, pro-inflammatory cytokine production, and activation of several enzymes which participate in the degradation of cellular components that pass through membrane micro ruptures, finally culminating in the activation of terminal cell death pathways.

Dystrophin is localized under the sarcolemma where it interacts with various glycoproteins forming the dystrophin-associated protein complex (DAPC). This multi-protein interaction underlies the dual function of dystrophin as a mechanical stabilizer, linking the ECM to the cytoskeleton, and a signalling platform that is essential for orchestrating, reactive oxygen/nitrogen species, membrane lipids and proteins e.g., cholesterol, caveolins, cavins, ion channels, and G protein-coupled receptors [52]. Myofibres lacking dystrophin are consequently sensitive to mechanical damage and have dysregulated cellular signalling which ultimately lead to cell death and consequent loss of tissue function. Increased intracellular calcium concentration, dysregulation of nitric oxide synthase (NOS), mitochondrial malfunction, inadequate anti-oxidant response, and several genetic modifiers are all implicated in the molecular pathophysiology of MD-CM [53,54,55,56] (see Figure 2). Overall, the causal mechanisms defining the final MD cardiac phenotype likely involve nuanced activation of known and novel pathophysiological pathways acting collectively on different cell types [57]. It becomes obvious that new, more powerful, and predictive patient-specific models are required to effectively address the unanswered questions concerning MD-CM pathophysiology and to identify novel therapies that might impact causal factors. Undoubtably, the recapitulation of certain fundamental hallmarks of MD-CM e.g., specific *DMD* mutation, lack of dystrophin expression, increased intracellular calcium [58,59,60,61,62,63,64,65,66,67,68,69] obtained with patient-specific cardiomyocytes derived from pluripotent stem cells will make significant additions, beyond the structural hypothesis of increased sensitivity to stretch-induced damage, to our knowledge of the pathomechanisms.

## 5. Current Pharmacotherapy

To date, there is no cure for MD since the currently available treatments are largely limited to the management of symptoms. However, the recent emergence of the novel mechanical support devices for breathing and non-disease specific drugs have prolonged life expectancy and improved the quality of life. Recently published guidelines for the management of cardiac disease in MD patients or CF acknowledge these novelties in treatment, however, there are still big variances in treatment protocols employed in different countries, cities and even specialized healthcare centres. With the prolonged patient life expectancy, MD-CM came to prominence as a new aspect of MD pathology that is quickly becoming the leading cause of death. Moreover, extended ambulation periods, as a consequence of improved skeletal muscle function, has been hypothesized to accelerate the development of MD-CM [70]. For this and similar reasons, it is fundamental to develop a multi-systemic approach to the management of this complex pathology that would integrate both cardiac and skeletal muscle treatment.

### 5.1. Corticosteroids

Treatment with corticosteroids is the current standard-of-care for MD patients with a recommended initiation age of between four to six years of age. There are numerous clinical trials where steroids have been shown to increase muscle strength and functionality [71,72], reduce the incidence of scoliosis [73] and prolong independent ambulation. Indeed, a large multi-nation study on 5345 DMD patients showed that the median age of ambulation loss in non-steroid treated patients was ten years old, compared with thirteen years old for the steroid-treated patients [74]. Corticosteroids also stabilize pulmonary function as patients receiving corticosteroids are ventilated later in life [75]. The beneficial effect of corticosteroid treatment is also extended to the heart. Some clinical trials show delayed progression of MD-CM (4% delay in the onset per year of treatment) [76], improved left ventricular fractional shortening [77], and reduced wall stress in comparison with non-treated patients [78]. The cardio-protective mechanism of steroids is still unclear, but may include: increased myogenic repair, stimulation of myoblast proliferation, sarcolemma stabilization and, reduced myocardial fibrosis and inflammation [74,76,79]. However, significant side effects include: decreased vertebral bone mass and increased in vertebral fragility, immunosuppression, weight gain, cataract formation and hirsutism [80,81]. In sum, steroids have many benefits for MD patients being advantageous for all major clinical outcomes [74]. Despite this, adverse events and unsuitability of steroids for long-term use must be considered especially among very young patients.

### 5.2. ACE Inhibitors

Angiotensin-converting enzyme (ACE) inhibitors are the most commonly prescribed treatment for MD after corticosteroids. These compounds are inhibitors of ACE that physiologically converts angiotensin-I to angiotensin-II, causing reduced circulating levels of the angiotensin-II. The use of ACE inhibitors delays the progression of cardiomyopathy, attenuates negative cardiac remodelling and improves cardiac function in MD patients [82]. Several preclinical and clinical studies support early treatment with ACE inhibitors. Rafael-Fortney et al. showed that the early treatment of *mdx* mice with the ACE inhibitor lisinopril and the mineralocorticoid-receptor antagonist spironolactone preserved cardiac function at 80% of normal, while only 40% of normal was reached when the treatment was delayed [83]. A clinical trial involving 28 DMD patients with normal systolic function treated with perindopril for three years showed a significant reduction of mortality after 10 years and advantages were also found for early compared to late drug administration [84].

### 5.3. Beta-Blockers

Beta-blockers (BBs) reduce sympathetic nervous system activity by interfering with the binding of catecholamines to β-adrenergic receptors. Despite not being the first choice for MD, BBs often combined with ACE inhibitors and diuretics in arrhythmic MD patients were shown to improve cardiac function and survival [71,72].

### 5.4. Angiotensin-II-Receptor Blockers

The angiotensin-II-receptor blockers (ARBs) are a good alternative for patients that cannot tolerate ACE inhibitors as they have similar efficacy, although they do not inhibit the breakdown of bradykinin. Therefore, they do not induce a dry cough or angioedema [85].

### 5.5. Mineralocorticoid-Receptor Antagonist

Mineralocorticoid-receptor antagonists, such as spironolactone, can be used in combination with ACE inhibitors and BBs in the treatment of MD-CM [86]. Spironolactone provided an anti-fibrotic effect that attenuated myocardial disease and improved myopathy in animal models of MD [83].

## 6. Benefits of Future Therapies

To date the majority of MD therapies undergoing research and development, and in some cases clinical trials, have focused on the treatment benefits for skeletal muscle function while omitting monitoring cardiac functions. Assessing the impact of a given therapy is generally easier in animal models of MD, however translation to humans has been beleaguered by failures. Therefore, gradually, as MD patients live longer and the issue of HF is increasing, a small number of trials included functional cardiac assessment among the study endpoints. For each future therapy (summarized in Table 1), we have included findings showing clinical benefit for the cardiac phenotype, however the greater part this data stems from animal-based studies.

### 6.1. Utrophin Upregulation

Utrophin is a protein that shares 80% of its sequence with dystrophin and has protein-binding properties similar to dystrophin. Unfortunately, these characteristics are not enough to fully substitute the absence of dystrophin, however it is reasoned that utrophin could partially compensate for dystrophin function. Therefore, mechanisms to induce the upregulation of utrophin are being investigated [87]. The hypothesis that utrophin could help cells with the lack of dystrophin developed through studying dystrophin/utrophin double knockout mice (*mdx*/*utrn*^−/−^) that presented with more severe skeletal muscle and cardiac pathology [87]. Furthermore, in *mdx* mice and DMD patients utrophin is overexpressed in skeletal muscle, thereby providing further evidence supporting the proposed hypothesis [87]. Many drugs have been evaluated in pre-clinical studies such as ezutromid (SMT C1100), a promising drug that was able to increase utrophin mRNA and protein levels in *mdx* mice [88]. Ezutromid passed a safety and tolerability phase I trial [89] and is now undergoing phase II trials. The main advantage of this treatment is that it can be universally prescribed to all MD patients independent of their underlying *DMD* mutation.

### 6.2. Stop Codon Read-Through Therapy

Gentamicin and negamycin are aminoglycoside drugs (antibiotics) that are able to overcome aberrant stop codons without affecting normal intended stop codons. In particular, they are able to bind specific sites in ribosomal RNA and impair codon-anticodon recognition at the aminoacyl-transfer RNA acceptor site [90] permitting translation of full-length proteins. About 10–15% of patients present a nonsense mutation and for this reason, gentamicin has been tested in DMD patients. However, study outcomes are inconclusive since, despite patients exhibiting decreased creatine kinase (CK) levels and low levels of de novo dystrophin expression, no other clinically meaningful endpoints changed in a significantly beneficial way [91]. Ataluren (PTC124) has been developed to address the need for a new drug capable of suppressing the premature termination of translation. It can induce ribosomal read-through of premature stop codons and its oral bioavailability is a distinct advantage [92]. Several clinical trials showed that Ataluren is well tolerated and some patient subgroups were positively affected by the treatment. In particular, the most responsive patient subgroup where those with a baseline six-minute walk test of 300 to 400 m [93]. The available data to support the therapeutic concept of mutation suppression, although clear clinical efficacy was not achieved. An additional trial (NCT03179631) that will examine the long-term efficacy and safety of Ataluren is currently planned and the results are imminent [93].

### 6.3. Viral Gene Therapy

A possible therapeutic strategy to treat DMD is the delivery of a dystrophin transgene to cells through a viral vector such as lentivirus [94], adenovirus [95] or adeno-associated virus (AAV). The capsid of all these vectors possess limited load capacity. Therefore, they are unable to carry the full *DMD* gene. Thus, truncated versions of the *DMD* gene (mini- or micro-dystrophin) have been developed to solve this issue [96,97]. The most promising vectors for MD gene therapy are AAVs because of their high transduction efficiency, persistence in the nucleus, tropism for skeletal and cardiac muscle, and low toxicity In order to increase safety, all viral sequences can be deleted from the original genome referred to as recombinant AAV (rAAV) [98,99,100,101,102]. In recent decades, several AAV serotypes were investigated for the treatment of DMD (see Table 1 for overview).

rAAV6 carrying micro-dystrophin was administered through single intravenous injection in young and adult *mdx* mice resulting in a widespread body transduction and dystrophin expression in skeletal muscles, diaphragm and heart [103,104]. rAAV8 and rAAV9 were successfully transduced in neonatal and adult mice through single intravenous administration, resulting in a widespread transduction of the heart and skeletal musculature [105,106]. Particularly, rAAV9 seemed to be more effective in terms of heart transduction efficiency and was successfully used to treat cardiomyopathy in neonatal and adult *mdx* mice using micro-dystrophin [107,109,152]. However, Bostick et al. showed that treated old *mdx* mice with end-stage dilated cardiomyopathy did not show the reversal of myocardial fibrosis while cardiac abnormalities were only partially corrected [108]. This result highlights an important limitation of this therapeutic approach in treating end-stage severe DMD cardiomyopathy. rAAV9 mini-dystrophin was also administered intravenously in neonatal golden retriever muscular dystrophy (GRMD) dogs, which lead to an inhomogeneous dystrophin expression in skeletal limb muscles and heart; muscular atrophy, contraction, and growth retardation were observed [110]. Positive results were also obtained by Yue et al. in juvenile DMD dogs using micro-dystrophin delivered through a tyrosine-engineered rAAV9 vector [111]. However, while the immunological response in murine models is limited, in larger animals [153] and humans [115,154] the reaction to viral vectors is still problematic. Furthermore, considering that repeated administration of rAAV vectors is required which can induce the generation of reactive antibodies to the vector capsid or transgene, i.e., the nascently expressed dystrophin [155,156,157]. Some of these issues could be partially surpassed by combining various elements: performing transient immunosuppression, selecting the most efficient and less immunogenic AAV serotype, optimizing the sequence of the transgene or reducing the viral load and choosing the proper route of administration [158]. In the context of MD gene therapy, high titres of virus are required to reach a proper transduction and consequent dystrophin expression in muscle cells. For these reasons engineered chimeric vectors were designed to optimize the transduction and reduce immunogenicity. Codon-optimized rAAV2/8 micro-dystrophin delivered intravenously to *mdx* mice and GRMD dogs resulted in successful transduction without immunological response [112,113]. Du et al. compared the transduction efficacy of five different hybrid vectors, namely rAAV2/1-5, administered in adult mice through intramyocardic injection. They showed that serotype 1 was the most efficient for heart transduction [114]. rAAV2.5 mini-dystrophin was delivered through intramuscular injection in the bicep muscle of six DMD boys (Phase I clinical trial, NCT00428935) which lead to effective gene delivery to the muscle cells with low immunological reaction to the viral vector; nevertheless, only very few dystrophin-positive myofibres were detected in two of the six patients [115,116]. A further complicating issue is the immunological response of the patient to the re-expression of dystrophin itself, due to the display of non-self epitopes as evidenced in preclinical and clinical trials [159,160]. In order to investigate the efficacy and safety of the AAV micro-dystrophin therapies to treat DMD, three clinical trials started recently in USA (i.e., NCT03368742, NCT03375164, and NCT03362502, see Table 1) [97].

### 6.4. Cell-Based Therapy

This promising therapeutic strategy involves the transplantation of cells that express normal dystrophin to a MD patient with the overall aim to restore the functionality of diseased muscle tissue. Healthy cells can be derived from the patient (autologous transfer) and delivered after ex vivo genetic modification (precision medicine) or can be obtained from an unaffected donor (allogenic transfer) in the form of an ‘off-the shelf’ product. A suitable cell type for this therapy should be able to migrate from blood to muscle, integrate into resident myocytes and self-renew to guarantee a long-term effect with minimal immunological impact. To date, several cell populations were demonstrated to have an active role in myogenesis and were studied for cell therapy application in MD (see Table 1). Satellite cells (SCs) are a heterogeneous Pax7+ cell population that resides in skeletal muscle, specifically within myofibres between the basal lamina and the sarcolemma. Normally, they are in the quiescent state, but they become active after injury and start to differentiate into myoblasts and proliferate; fusing together and to existing myofibres in the attempt to repair the muscle [159,161]. Myoblast engraftment has been proven to be inefficient because of limited migration and poor in vitro survival in human MD patients [161]. Montarras and colleagues purified a population of satellite cells Pax7+/CD34+/CD45-/Sca-1-derived from the diaphragm of Pax3GFP/+ mice through FACS. After grafting into irradiated TA muscle of *mdx nu*/*nu* mice, these cells contributed both to muscle repair and the restoration of the SC compartment [117]. Another study followed this approach obtaining restored dystrophin expression, improved contractile function, and renewal of the SC pool on normal and *mdx* mice [118]. Curiously, regenerative capacity and myogenic potential are compromised if cells are expanded in vitro before engraftment [117]. This represents an obstacle from a clinical standpoint, especially in an autologous transplantation scenario that is the ideal approach because an immunological response is minimized. Another population of skeletal muscle resident cells, the muscle-derived stem cells (MDSCs), which are distinct from SCs, exhibit multipotency and self-renewal capability [119,162]. In particular, they maintain their proliferative and myogenic potential when cultured in vitro, with proliferation being retained following differentiation into other lineages (e.g., muscle, neural, and endothelial). MDSCs can also migrate through the vasculature and integrate effectively into the host tissue, all features that make them clinically suitable [119,163]. Subpopulations of MDSCs were isolated in order to achieve high levels of transplantation efficiency and dystrophin expression after transplantation into *mdx* mice [119,120]. Several works demonstrated the contribution of non-myogenic cell populations to muscle regeneration. Bone marrow-derived stem cells (BM-MSCs) injected into irradiated mice were shown to reconstitute the skeletal muscle stem cell niche, regenerate muscle fibres, and partially restore dystrophin expression [121,124,125]. A subpopulation of MDSCs of haematopoietic origin, called muscle side population cells (m-SP) were demonstrated to have myogenic potential, to restore dystrophin expression in *mdx* mice, to migrate from blood stream to muscles and to differentiate into functional SCs after transplantation [121,122,123]. Mesoangioblasts, vascular resident mesodermal progenitors, were transduced with a lentivirus expressing micro-dystrophin and restored dystrophin expression in GRMD dogs [126]. Mesoangioblasts were also employed to transfer an artificial chromosome carrying a whole dystrophin to *mdx* mice that resulted in an amelioration of the dystrophic phenotype lasting up to eight months, thus highlighting the strong clinical potential of this approach [127]. Pericyte-derived cells were administrated to severe combined immune deficient-X-linked *mdx-scid*-mice resulting in a high number of dystrophin-expressing muscle fibres [128]. Human DMD muscle-derived CD133^+^ stem cells were corrected ex vivo through lentiviral exon skipping and then engrafted into *mdx-scid*-mice which showed improved muscle functionality and dystrophin expression [129]. Autologous transplantation of muscle-derived CD133^+^ cells was then successfully performed in phase I clinical trial involving eight DMD boys that assured treatment safety and some degree of improvement in markers of skeletal muscle function [130]. It is also possible to derive myogenic progenitors from embryonic stem cells (ESCs) and induced pluripotent stem cells (iPSCs) by conditional expression of Pax7 and engraft the resulting cells into the TA muscle of NSG/*mdx* mice that produced dystrophin-positive myofibres and contributed to the SC pool [131]. In a therapeutic perspective, this result suggests that patient-derived iPSCs can be differentiated in vitro into muscle precursors and administrated to the patient without an immune response. Dystrophin expression can be restored by modifying the genome of iSPCs in vitro through splice correction. Li and colleagues employed TALENs and CRISPR-cas9 to induce exon skipping, frameshift, and exon knockin in human DMD iPSCs and then differentiated the edited iPSCs into skeletal muscle cells, where dystrophin expression was observed [164]. Although intramuscular administration is currently the most efficient way to perform cell delivery [165], this approach implies that each muscle must be treated individually and that muscles like heart and diaphragm are excluded. Therefore, a systemic delivery is preferable over direct delivery to isolated sites in order to foster a widespread effect and reduce the number of injections that DMD patients must undergo. 

### 6.5. Antisense Oligonucleotides (AONs)

AONs are small pieces of modified nucleic acids complementary to a target pre-mRNA splicing site or to a specific splicing regulatory element (i.e., splicing enhancer/splicing silencer) [166]. AONs are used to restore the correct dystrophin open reading frame, skipping a target exon in patients that carry deletions, duplications, or small mutations that alter the *DMD* reading frame. They are also applicable to exon skipping of in-frame nonsense mutations [167]. The aim in both cases is to induce the production of a partially functional dystrophin protein [168]. This treatment has the theoretical potential to treat about 83% of DMD patients [169]. The shortcoming of this approach is reflected in the fact that each patient carries a different mutation requiring different exon skipping. Thus specifically designed functional AONs are required for each unique *DMD* mutation. Consequently, only the most common mutations have been investigated, namely exon 51, 45, and 53. It is certain that new AONs targeting other exons will be needed, however, it must be considered that for most other exons lying outside the hotspot region (exons 45 to 53) the applicability is <1% of patients, and this poses challenges to the clinical development of AONs [170]. Since exon skipping is a mutation-specific approach and in some patients the reading frame can be restored by a single exon skipping other patients might require double exon skipping that requires the combination of two AONs. This is feasible in the in vitro setting where the transfection efficiency on cultured cells is very high (close to 100%) [171]. However, double exon skipping in vitro is remarkably less efficient, so further investigation is required [168]. Another important limiting aspect of the exon skipping approach is the lack of production of a fully functional dystrophin protein e.g., when mutations abolish all actin-binding domains, the dystroglycan binding domain or when the deletion involves 36 or more exons [172,173].

From a chemical structure point of view, AONs can be divided into three groups, each one with different pharmacokinetic, pharmacodynamic, and safety profiles. Firstly, the phosphorodiamidate morpholino oligomer backbone is based on phosphorodiamidate morpholino oligomers (PMOs) that have a neutral charge. These types of AONs showed some promising results in the clinic, but have a poor systemic delivery record and are unable to restore dystrophin expression in the heart [132]. 2′O methyl phosphorothioate (2OMePS) are a second AON group that are negatively charged which leads to a high level of plasma protein binding that increases their half-life [136]. They have different chemical structures in comparison to PMOs. However they showed similar efficacy in the first clinical trials [132,137,174]. Lastly, the third group of AONs, tricyclo-DNA, were developed in an attempt to overcome the poor systemic delivery of the two previous AONs. These new oligonucleotide analogues have three additional carbon atoms between C5′ and C3′ that functionally increase hydrophobicity, nuclease resistance, and RNA affinity [138]. This AON category has shown an important preclinical success. Dystrophin was restored following treatment both in skeletal muscle and the myocardium in *mdx* mice and at lower doses in comparison with 2OMePS and PMOs indicating the superiority of Tricyclo-DNA for exon skipping [138]. In an effort to improve the delivery capacity of AONs without changing the chemical backbone, another possible strategy is the conjugation of neutral AONs (e.g., PMOs) with short peptide sequences called ‘cell penetrating peptides’ (CPPs). Positively charged arginine-rich peptides conjugated to PMO (PPMO) have been tested in *mdx* mice demonstrating: enhanced uptake by most tissues, including the heart and increased levels of exon skipping and dystrophin expression [175]. Unfortunately, these types of arginine-rich peptides were poorly tolerated in higher animals and humans because of renal toxicity [175]. More recently, Pip peptides were developed and tested in the *mdx* mouse. In particular, systemic administration of the most potent Pips, the Pip 5 and Pip 6 series, resulted in exon 23 skipping with dystrophin protein production in skeletal and cardiac myocytes and improvements in skeletal muscle strength and cardiac functions [139,140,176]. These newly developed CPPs seem to be well tolerated in *mdx* mice, but they contain many arginine residues, thus possible side effects need to be considered. CPPs have a cationic nature and for this reason, they are not suitable for delivery of negatively charged 2OMePS because they have a strong tendency to aggregate. For this type of backbone, a 7-mer peptide was identified (through phage display experiments) which increased AON uptake and effect in the heart after systemic administration in *mdx* mice [141]. Tissue-specific homing peptides are undergoing investigation to improve the uptake of AONs. These peptides are identified by phage display biopanning. An example of a muscle-targeting peptide is ASSLNIA [177] which when tested in conjugation to PMOs targeting *DMD* resulted in improved dystrophin restoration in skeletal and heart muscles, and functional improvement of the dystrophic phenotype in *mdx* mice [178]. 

Two AONs targeting exon 51, have reached the clinic, namely drisapersen (PRO051, GSK2402968) and eteplirsen (AVI-4658). Although drisapersen, a 2OMePS-modified AON did not get FDA or EMA approval (the EMA application was withdrawn by the applicant before evaluation completion); eteplirsen had more success being rather controversially given accelerated approval by the FDA at the end of 2016. Regarding eteplirsen, multiple clinical trials demonstrated that dystrophin protein levels were increased, however, a clear efficacy was not demonstrated. Indeed, dystrophin expression was used by the FDA as a surrogate endpoint, which they accepted as proof of efficacy [133]. The EMA position is quite different, increased dystrophin expression is not considered a surrogate measure of efficacy as stated in published EMA guidelines on DMD. Despite this and considering the urgent need for a treatment, eteplirsen is currently under EMA review. In 2018, Shimizu-Motohashi et al. published a meta-analysis that considered five randomized controlled trials (RCT) on exon-skipping drugs (eteplirsen and drisapersen) involving 322 DMD patients and assessed their efficacy and limitations. The results of the meta-analysis showed no significant overall effect of exon-skipping treatment. In particular, no significant difference was found in the distance covered by treatment and placebo groups in the 6-min walking test (6MWT) from baseline to week 24 of treatment period [134]. Furthermore, treatment with drisapersen was associated with a significantly higher number of patients with injection site reaction and renal toxicity in comparison to patients treated with placebo or etelprirsen, which had no significant side effects [134]. The FDA has mandated a post-marketing RCT to prove the efficacy of eteplirsen, with results to be reported by 2021 [135].

### 6.6. Possible Other Treatments and Drug Repositioning

Membrane tears are thought to play a role in DMD pathophysiology and for this reason compounds like the synthetic copolymer poloxamer P188, a molecule that stabilizes the sarcolemma, have been investigated [142]. Yasuda et al. showed that the administration of P188 to *mdx* mice during dobutamine treatment (mediated-stress protocol) prevented the development of HF. Another available compound is MG53, an essential component of the membrane repair cascade in striated muscle. Injection of recombinant MG53 prevented exercise-induced skeletal muscle damage in *mdx* mice [143]. 

Sildenafil enhances nitric oxide (NO) cyclic guanosine monophosphate (cGMP) signalling resulting in increased cGMP and vasodilation. Studies on *mdx* mice treated with sildenafil showed improved contractile performance, cardiac metabolism, and sarcolemma integrity [144]. In addition, the treatment with sildenafil also improved diaphragmatic muscle strength and enhanced respiratory function [145]. However, these positive results have not translated to humans and two clinical trials have been prematurely terminated due to futility and the absence of clear clinical benefit [146].

Losartan and pirfenidone are two compounds with anti-fibrotic properties capable of inhibiting TGF-β expression and consequently reduce fibrosis and improve cardiac function [147,148]. 

Myostatin is a growth factor that belongs to the superfamily of TGF-β signalling molecules and its expression is predominant in the skeletal muscles [179]. Myostatin is a physiological antagonist of insulin-like growth factor-1 (IGF-1) that mediates muscle atrophy and inhibits skeletal muscle growth at all developmental stages [149]. Several in vitro studies showed that treatment with myostatin inhibits the proliferation and differentiation of muscle precursors [180,181,182]. Studies on *mdx* mice suggest that blockade of myostatin signalling ameliorates the dystrophic process by enhancing muscle regeneration and growth, and by decreasing fibrosis [149]. Unfortunately, clinical trials did not show positive results and significant side effects were reported e.g., nosebleeds, gum bleeding, and dilated blood vessels within the skin. In addition, it is not well established if the tested compounds have specificity of action in muscles. The principal compounds tested are MYO-029 that is an anti-myostatin monoclonal antibody [150] and ACE-031, a soluble form of activin type II receptor B (ActRIIB) (physiologically, the binding of the active form of myostatin with this receptor inhibits muscle growth) [183,184]. In conclusion, myostatin blockade could be a possible therapeutic approach for MD patients, but the development of skeletal muscle-specific ligand is required in the future. 

Urocortin is a corticoid releasing factor (CRF) receptor agonist; beneficial effects on skeletal muscle structure and function in *mdx* mice have been reported [151].

## 7. Radical or Conservative Disease Management

Corticosteroids, the most relevant class of drugs for DMD, have profound effects on the disease course. Evidence from early RCTs indicate that corticosteroid therapy in DMD enhanced short term muscle strength and function, and strength for up to two years [185]. Subsequently, the long-term effects of steroid therapy—such as prolonged ambulation, delayed scoliosis, and preservation of cardiopulmonary function—have also been recognized. Additionally, steroid administration may also have a positive impact on LV function in patients with DMD, although the exact mechanisms underlying this process remain unclear. The DMD Care Considerations Working Group identified ACE inhibitors and ARBs as first-line therapies in patients with DMD with LV dysfunction [186]. ACE inhibitors significantly reduced mortality and hospitalization rates in these patients [187]. However, the use of BBs in DMD patients has been tested only in small-sized studies [188]. Viollet et al. demonstrated that treatment with ACE inhibitors and BBs, when properly titrated according to dysfunction and symptom progression, may delay the progression of MD-CM [189,190]. While there is no current consensus regarding the age at which these treatments should be initiated, it is recommended to begin treatment as early as possible in DMD patients with initial signs of myocardial impairment.

COX-inhibiting nitric oxide (NO) donors represent a new pharmaceutical category recently introduced in the treatment of DMD patients. This class of drugs have a structure similar to non-steroidal anti-inflammatory drugs, but with a higher NO transporting capability, thus decreasing inflammation in both skeletal and cardiac muscles [191]. Novel therapies, targeting *DMD* mutations, are under preclinical or clinical evaluation, including viral vector-based gene therapy and AONs for exon skipping.

Heart transplantation has not been considered an appropriate treatment option for DMD patients, because of pulmonary and skeletal muscle impairment [192]. However, a possible treatment for end-stage HF in these patients is the use of a left ventricular assist device (LVAD) as a destination therapy [193,194]. In real-life clinical scenarios, the availability of various implantable devices and a relative inadequacy of current guidelines complicate clinical decision-making when caring for MD patients with end-stage HF and severe LV dysfunction [195,196,197]. There is a paucity of studies addressing the use of LVADs in patients with DMD [198,199,200]. Therefore, a careful preoperative multidisciplinary evaluation of candidate patients is warranted [197]. Likewise, postoperative complications—including respiratory failure, bleeding, stroke, and arrhythmias—along with rehabilitation outcomes still need to be assessed by long-term survival studies. Currently, considerable ethical issues remain open regarding the use of LVAD as a palliative therapy for patients with no other therapeutic options [197,198,200,201]. Therefore, further prospective studies with a longer follow-up period are necessary to fully legitimize this strategy for MD patients.

## 8. Disease Modelling

The description of the genetic and the molecular basis of dystrophin-associated pathologies have been gained mainly through the analysis of muscle biopsies from healthy and diseased donors or by performing experiments in animal models. The use of animal models has provided a valid alternative to human tissue as the latter has limited availability and rising ethical concerns for both. Unfortunately, due to the lack of accessible cardiac tissue from MD patients and from healthy controls, most of the data on the molecular, cellular, and functional mechanisms of MD-CM have been collected from expensive, low throughput animal models that do not always fully recapitulate human pathophysiology. While in vitro models remain an essential step for the preclinical validation of new drugs, their high complexity and the presence of compensatory effects impede the decryption of molecular pathways and cellular mechanisms. The potential of in vitro studies could be augmented by the application of in vitro high throughput models. The ability to generate diseased cardiomyocytes from human induced pluripotent stem cells (CMs-d-iPSCs) has realized a human cellular model available for in vitro characterization of several cardiac pathologies [202]. Despite their immature state, MD patient-specific CMs-d-iPSCs represent the only cell type that allows the study of the signalling, mechanical and electrical properties of human cardiomyocytes in the context of the specific genetic background associated with a disease. The applicability of this cell type is limited only by the lack of systems able to accurately replicate the interplaying in vitro environmental cues. To address this, the design of in vitro models able to simulate the natural regenerative processes, recreate the progression of the pathology and evaluate the efficacy of possible treatments, is considered the new frontier in the field. The objective of this section is to discuss the relevant parameters for the design of such in vitro models; starting from cell source and continuing to define the environmental cues needed to support cell maturation. Particular attention is focused on recapitulation of the in vitro and pathophysiological scenarios.

### 8.1. Cell Source

While the isolation of myocytes from muscle biopsies for experimental use from MD patients raises major ethical concerns, the use of patient-specific iPSCs [203] is a valid alternative as they have relatively lower physical repercussions and ethical issues. Indeed, this technology offers the possibility to obtain pluripotent human cell lines, derived from skin or blood of healthy and diseased adult donors, and differentiate them into several tissue-specific and disease-relevant cells. Thus patient-specific iPSCs not only lessen ethical issues, but, even more interestingly, provide the unique chance to study the effect of the same mutation on different tissues and organs, distinguish levels of severity, study the response to treatment and, finally, open the way to a precision medicine approach to MD-CM treatment [204].

Pluripotency is, by definition, the main feature of iPSCs. Thus making them very adaptable and very sensitive to external stimuli [205,206,207,208]. Consequently, the design of in vitro experiments is paramount, requiring a deep deliberation regarding the definition of the right balance between simplicity and thoroughness. Indeed, it has been widely demonstrated that 2D culture on plastic plates is unable to support the differentiation of iPSCs into mature somatic cells, in particular skeletal [209] and cardiac myocytes [210,211], thus limiting the significance of possible treatment evaluation protocols and the understanding of the relevant signalling pathways. 

### 8.2. Environment Matters

The development of mature cells is a prerequisite for the generation of in vitro models descriptive of the in vitro scenario. By implementing a biomimetic approach, cell growth and differentiation could be supported through the application of relevant chemical and mechanical stimuli. iPSC potency and their capability to undergo differentiation into mature somatic cells is supported by biochemical and mechanical stimuli, mediated by extracellular matrix (ECM) and neighbouring cells. Cell–matrix adhesion and cell–cell interaction have a demonstrated effect on cell morphology and the expression of pluripotent genes [212] governed by mechanotransduction pathways [206,207] and several in vitro models have been proposed to test mechanosensing dependent responses. In general, culturing iPSCs on substrates coated with native ECM components significantly increases cell maturation levels. Skeletal and cardio myocyte differentiation are supported by culture on collagen [211], laminin [213], fibronectin [205,213], vitronectin [214,215], or on fibroblast-derived matrices [206]. ECM composition has special relevance for MD patients both in vitro and in vitro as it has been demonstrated that the lack of dystrophin protein induces a disruption of the dystrophin glycoprotein complex, negatively impinging membrane stability and actin cytoskeleton engagement. This induces compensatory effects by modifying secretion of ECM components, e.g., glycosaminoglycans and collagens [216]. Such modifications of ECM composition affect the mechanical and geometrical features of the micro-environment surrounding cells, possibly further regulating the activation of cell machinery in a pro-pathological direction, as widely demonstrated in other scenarios [212,217,218]. 

Several works examined the role of matrix rigidity on cell reprogramming [211,217,219,220,221], underlining how pluripotent stem cell differentiation and further maturation could be directed by substrate stiffness [206,207,211,217,222]. In particular, a positive effect on cardiac commitment has been demonstrated by growing and differentiating iPSCs on substrates with stiffness resembling that of healthy cardiac tissue [209,223,224]. These approaches enhance metabolic maturity, ion handling capability, sarcomeric protein subtype, cardiac troponin T expression [217] and force generation [222]. Given the modifications in local ECM rigidity observed in skeletal and cardiac tissue in DMD patients, the study of the pathways leading to these matrix modifications and the effects on the evolution of the pathology are extremely relevant for future investigations. Mechanosensing pathways respond not only to substrate rigidity but also to geometric stimuli. In vitro cardiac and muscle tissues are characterized by cell alignment and anisotropy, and the effect of this geometrical organization on the maturation of iPSC-derived cells has been demonstrated in vitro by using micro- and nano-structured substrates, shown to support the formation of cell-to-cell junctions, building foundations for the generation of functional grafts, showing a contractile phenotype, increasing parameters such as beating rate and tissue-specific protein arrangements (e.g., sarcomeric α-actinin, connexin 43 and troponins) [209,225,226].

While 2D in vitro models are valid tools to construe specific pathways, especially under static conditions, they fail to recapitulate the complexity of native tissues. In particular, their limitations become evident in describing tissues, such as skeletal muscle and the heart, which characteristically possess a wide range of motion and dynamic forces that are essential triggers for specific cell differentiation and matrix deposition required for tissue development and functionality [227]. Nevertheless, moving to a 3D system does not only imply the design of 3D scaffolds, fusing the controlled chemical, mechanical, and geometrical features optimized by the 2D experience, but calls for the organization of a hierarchy. Indeed, in the specific case of skeletal and cardiac tissues, mass transport diffusion, and spatial limitations should be overcome and electric stimulation should be simulated. Thus, several micro-devices have been designed and validated, following an ‘organ/lab on chip’ approach [228,229]. The use of such systems offers the advantage to overcome and/or support complex [230,231] co-culture to recreate vascularization and innervation, respectively using artificial perfusion, e.g., micro-channels, and/or electrical stimulation [232,233,234]. Further development of these systems could be reached by coupling them with novel bioprinting methods, allowing simulation of complex 3D structure, thus the use of either macro- or micro-bioreactors would offer the chance to perform monitored and standardized in vitro culture protocols leading to the generation of 3D engineered mature tissue grafts. These would allow finding a correlation between imposed culture conditions and resulting tissue functionality. The optimization of methods, compatible with meaningful read out parameters that provide a quantitative description of tissue or disease development, such as electrophysiological measurements and tissue-generated forces, is still an open question [235,236,237]. Indeed, from the translational point of view, these data could be more relevant than the measurement of indirect biochemical parameters e.g., dystrophin expression. A more precise and quantitative evaluation of the maturation level of the cells and/or grafts is fundamental to solve a central discussion point in the validation of in vitro models ´How good is good enough?´. Indeed, it could provide a method for the evaluation of the optimal maturation level needed to reproduce the relevant critical aspects of the physio/pathological scenario to answer a specific question e.g., drug efficacy.

In the future, the generation of model systems allowing the culture of iPSCs in a complex 3D highly structured and controlled environment could allow the evaluation of the responses to mechanical, electrical, and chemical stimuli. This approach would, therefore, guarantee the double benefit of better recapitulating pathophysiological processes and to evaluate the efficacy of novel treatments e.g., pharmacological compounds. Indeed, it has been demonstrated, also for DMD pathology [238], that culturing iPSCs in a tissue-specific environment allows better stratification of the differences between healthy and pathological subjects, allowing a more efficient chemical compound screening. Moreover, coupling 3D micro-bioreactors and iPSC technology, could allow the parallel study of the effect of the same treatment on different regions affected by a pathology, e.g., differentiating iPSCs from patients affected by different variants of the same pathology into both mature skeletal and heart muscle, paves the way to the personalized medicine approach [239].

## 9. Future Perspectives

In the era of personalized medicine there is much hope for continued improvement of treatment options for MD patients ranging from gene therapy to cell therapy-based products. However, the MD community, as a whole, must continue strieving for meaningful treatment advances while simultaneously managing the epectations placed upon emerging therapies and research. Patient-specific cells cultured in optimized disease model platforms offer the possibility of bespoke, predictive therapies for MD patients—the veritable ‘holy grail’ in MD-CM treatment.

## Figures and Tables

**Table 1:** Ongoing therapies to treat DMD.

Treatment	Research Stage	Methods and Results	References
**Utrophin up-regulation**			
**SMT C1100**	Preclinical—*mdx* mice. Clinical, Phase I—DMD children	Adult *mdx* mice were daily administered with SMT C1100 (ezutromid, 50 mg/kg), an utrophin modulator, for 4 weeks: increased mRNA and protein levels of utrophin; reduction of skeletal muscle inflammation and fibrosis; protection form exercise-induced injury. In a clinical trial (NCT02383511), 12 DMD patients were treated with SMT C1100 (50 and 100 mg/kg bid or 100 mg/kg tid): assess safety and tolerability.	[88,89]
**Stop codon read-through therapy**			
**Ataluren**	Clinical, Phase III—DMD boys	In a clinical trial (NCT01826487), Ataluren (PTC124) was administered to DMD boys with a nonsense mutation for 48 weeks (40 mg/kg daily): assessed tolerability; positive effects, particularly on the subgroup of patients with baseline 6MWD between 300 m and 400 m (least square mean difference of 42.9 m; *p* = 0.007). Another trial (NCT03179631) started to examine long-term effects of Ataluren in 250 DMD 5-year and older patients.	[93]
**Viral gene therapy**			
**Lentivirus**	Preclinical—*mdx*	Micro-dystrophin IM injection into TA muscle of neonatal *mdx* mice: stable expression (20–25% of CSA) of dystrophin up to 2 years; ameliorated pathophysiology but no protection from c.i. injury; transduced both myofibres and satellite cells that contributes to muscle regeneration.	[94]
**‘Gutted’ adenovirus**	Preclinical—*mdx*	IM injection of full-length dystrophin cDNA into TA muscle of 1-year-old *mdx* mice: dystrophin expression 1 month after injection (25–30% of CSA) and ≈ 40% correction of susceptibility of muscles to c.i. injury.	[95]
**rAAV6**	Preclinical—*mdx/utr^-/-^* and *mdx* mice	Single IV administration of micro-dystrophin in 1-month-old *mdx/utr^-/-^* and 20-month-old *mdx* mice. In young mice: body-wide dystrophin expression 1 year post-injection; efficient transduction of diaphragm with improved resistance to c.i. injury; heart transduction and normal heart mass but no alteration of myocardial performance index; increased size of TA muscle, peak force production and resistance to c.i. injury. Increased body mass and extended lifespan of treated mice. In old mice: widespread expression of dystrophin 4 months after injection in skeletal muscle, diaphragm and heart; increased resistance to c.i. injury of diaphragm; increased peak force production of TA muscle.	[103,104]
**rAAV8**	Preclinical—normal mice	Single IV and IP administrations of the viral vector in neonatal and adult mice: efficient gene transfer in skeletal muscles and heart.	[105]
**rAAV-rh74**	Clinical trial, Phase I/II—DMD infants and children	One clinical trial (Jerry R. Mendell, NCT03375164, drug name rAAV-rh74.MHCK7.Micro-dystrophin) started in USA to assess the safety of micro-dystrophin delivery with rAAV-rh74 in very young dystrophic patients (from 3 months to 7 years). Only ambulatory patients with frameshift or nonsense mutation within exon 18–58 are recruited.	[97]
**rAAV9**	Preclinical—normal mice, *mdx* mice, GRMD dogs and DMD dogs. Clinical trial, Phase I—DMD children and boys	Single IV administration of the viral vector in six to eight-week-old mice resulted in an efficient heart transduction. Neonatal and 6-week-old *mdx* mice were treated with micro-dystrophin through single IV administration: heart transduction and improvement of cardiac pathology. Single IV administration of micro-dystrophin in 16 to 20-month-old female *mdx* mice with severe cardiomyopathy: efficient cardiac transduction after 2–8 months and improvement of cardiac pathology. Treatment of >21-month-old *mdx* mice with micro-dystrophin revealed strong dystrophin expression in the heart but only partial correction of ECG abnormalities and no improvement in cardiac fibrosis. Single IV administration of mini-dystrophin in neonatal GRMD dogs: efficient limb muscles, diaphragm and heart transduction after 16 weeks (from 15 to 100% dystrophin-positive myofibres); inflammatory myopathy, contractures and growth retardation were observed. Micro-dystrophin was administered to 2-month-old DMD dogs through single IV injection of tyrosine-engineered vector carrying micro-dystrophin; immunosuppression was performed: widespread transduction in skeletal muscles, diaphragm and heart after 16 weeks without adverse reactions. Two clinical trials started in USA to assess the safety of systemic micro-dystrophin delivery with AAV9 vector. The first (Solid Biosciences, NCT03368742, drug name SGT-001) recruits both ambulatory and non-ambulatory patients, the second (Pfizer, NCT03362502, drug name PF-06939926) recruits only ambulatory patients. In both cases, children and adolescents with any *DMD* mutation are recruited.	[97,106,107,108,109,110,111]
**rAAV2/8**	Preclinical—*mdx* mice and GRMD dogs	IM and IV administrations of codon-optimized micro-dystrophin in neonatal and adult *mdx* mice: dystrophin expression in both heart and skeletal muscles after systemic administration (12 weeks post injection); improved muscle function and protection from c.i. injury; no immunological response was observed. Juvenile GRMD dogs were treated with IV and LR (i.e., forelimb) administrations of canine micro-dystrophin without immunosuppression: high protein expression (50% on average) in the treated limb and recover of function after LR perfusion; significant amelioration of the clinical status and gait quality (up to 2 years) following IV injection. No data regarding heart pathology.	[112,113]
**rAAV2/1-5**	Preclinical—primary neonatal and adult mice CMs, primary human CMs and adult mice	Murine and human CMs were cultured in vitro and infected with five rAAV serotypes (1 to 5). In detail, murine neonatal CMs were cultured for 7 days after infection while primary human and murine CMs only for 72 h. IC injection of five serotypes of the viral vector was performed in 10-week-old mice. Serotype rAAV1 has shown a good cardiac transduction efficacy in vitro (12 and 10% positive CMs in murine and adult CMs respectively) and also in vivo (40% positive CMs 1 month after infection).	[114]
**rAAV2.5**	Clinical trial, Phase I—DMD boys	Six DMD boys were treated through IM administration of mini-dystrophin in the bicep muscle (Jerry R. Mendell, NCT00428935). Only few dystrophin-positive myofibres were detected in two patients and T cell-mediated immune response against mini-dystrophin and the viral capsid was observed.	[115,116]
**Cell-based therapy**			
**Satellite cells (SCs)**	Preclinical—*mdx* and *mdx nu/nu* mice	Isolation of SCs from the diaphragm of Pax3^GFP/+^ mice through FACS and injection in the irradiated TA muscle of 3-week-old *mdx nu/nu* mice: dystrophin expression three weeks after transplantation and contribution to the satellite cell pool. Reduced efficiency after culturing was observed. A subpopulation of stem cells, namely skeletal muscle precursors (SMPs), were purified through FACS from normal mice and engrafted into limb muscles of *mdx* mice: high efficiency (up to 94% of engrafted myofibres); restored dystrophin expression; improved muscle functionality and renewing of the satellite cell niche. No heart data available.	[117,118]
**Muscle-derived stem cells (MD-SCs)**	Preclinical—normal, *scid/bg* and *mdx* mice	Hindlimb IA injection of purified CD34^+^/Sca-1^+^ MD-SCs isolated from newborn mice into 3-month-old *mdx* mice: adhesion to the endothelium of muscle microcirculations; migration to limb muscles and dystrophin expression. A CD34^+/-^/Sca-1^+^/c-kit^-^/CD45^-^ MD-SC population was purified from mice relying on their adhesion behaviour and transplanted into the limb muscles of 6–8-week-old *mdx* mice: dystrophin positive myofibres were detected 90 days after implantation; proliferation, self renewal capability and multipotency were assessed both in vitro and in vivo. Muscle side population (m-SP) cells were obtained from normal mice and injected IV into irradiated female *mdx* mice: up to 6% dystrophin positive myofibres after 30 days. FACS/Hoechst-purified m-SP cells were obtained from transgenic mice and injected into the regenerating TA of *scid/bg* mice: differentiation into myocytes and satellite cells and fiber regeneration in the injured site. Purified m-SP cells were isolated from donor mice and injected into damaged TA of irradiated mice: CD45^+^ m-SP integrated into regenerating myofibres. No heart data available.	[119,120,121,122,123]
**Bone marrow-derived stem cells (BM-MSCs)**	Preclinical—normal, *scid/bg* and *mdx* mice	Haematopoietic stem cells from normal mice were administered to irradiated female *mdx* mice through IV administration: up to 4% dystrophin positive myofibres detected 12 weeks after transplant in the TA muscle. BM-MSCs (GFP^+^) from donor mice were injected IV into irradiated mice: GFP^+^ cells were found into the TA muscle after 6 months, occupying the ablated satellite cell niche; regeneration of muscle fibres after exercise-induced damage. BM-MSCs cells from transgenic mice were injected IV into irradiated *scid/bg* mice with chemically induced TA muscle degeneration: migration of BM-MSCs to the injured site; myogenic differentiation and regeneration of the damaged fibres. No heart data available.	[121,124,125]
**Mesoangioblasts**	Preclinical—*scid/mdx* mice and GRMD dogs	Mesoangioblasts from *mdx* mice were genetically corrected with human artificial chromosome carrying the whole human dystrophin genetic locus; cells were lentivirally transduced with MyoD and nLacZ and transplanted into 4-month-old *scid/mdx* mice through IM and IA delivery: dystrophin positive myofibres; contribution to satellite cell pool and improved muscle function up to 8 months after administration. GRMD dogs received wild-type heterologous and genetically-corrected autologous mesoangioblasts after IA delivery: increased dystrophin expression; improved muscle function and mobility; low immune reaction. No heart data available.	[126,127]
**Pericytes**	Preclinical—*scid/mdx* mice	Pericyte-derived cells were isolated from muscular biopsies of human healthy and dystrophic subjects and transplanted into irradiated *scid/mdx* mice: colonization of host muscle and dystrophin expression. No heart data available.	[128]
**CD133^+^ stem cells**	Preclinical—*scid/mdx* mice. Clinical, Phase I—DMD boys	Blood- and muscle-derived CD133^+^ cells were isolated from human dystrophic subjects, genetically corrected to re-express dystrophin and injected into *scid/mdx* mice: restored dystrophin expression and recovery of muscle function. In a clinical trial, eight DMD boys were treated with autologous muscle-derived CD133^+^ cells through injection in the ADM muscle: increased number of capillaries per muscle fiber; switch from slow to fast myofibre type; assessed safety of the procedure; no alterations in the cardiac dimensions and function.	[129,130]
**iPSCs**	Preclinical—*NSG/mdx* mice	Pax7-derived myogenic progenitor cells were generated from human ESC and iPSC cells and injected in the TA of adult *NSG/mdx* mice: stable engraftment (up to 11 months); dystrophin expression; enrichment of satellite cell niche; improved muscle strength. No heart data available.	[131]
**Antisense oligonucleotides (AONs)**			
**PMO**	Preclinical—*mdx* mice. Clinical, Phase IV	Weekly IV injections of PMO (skipping exon 23) into adult *mdx* mice: body-wide dystrophin expression in the skeletal muscles (>70% in quadriceps and gastrocnemius after seven injections), although low in the diaphragm and absent in the heart; transcript lacking exon 23. Another AON targeting the exon 51, namely eteplirsen (AVI-4658) was approved by FDA in 2016, although its effectiveness for the treatment of DMD remains controversial.	[132,133,134,135]
**2OMePS**	Preclinical—*mdx* mice. Clinical, Phase III	Young to adult *mdx* mice were treated with IV injections with 2OMePs together with tryblock copolymer F127: exon 23 skipping confirmed; induced dystrophin expression in skeletal muscles, notably in the diaphragm, but not in the heart; more effectiveness in older mice; no toxic effects. In a Phase I/II clinical trial (NTR1241) 12 DMD patients were treated with PRO051 (drisapersen) (0.5, 2, 4 and 6 mg/kg, SC weekly injections) for 5 weeks to induce exon 51 skipping; extension of the treatment for 12 weeks (6 mg/kg per week): assessed safety; detectable exon 51 skipping; restored dystrophin expression; positive effects in 6MWD. A randomized, placebo-controlled Phase III trial (NCT01254019) in 186 ambulant boys aged ≥5 years again evaluated the long-term efficacy and safety of subcutaneous drisapersen (6 mg/kg/week, 48-week); a favourable response time in the 6MWD was recorded for drisapersen at 48 weeks with further analysis concluding suggesting drisapersen could specifically benefit a patient subpopulation with a milder disease impairment. Notably, drisapersen did not get FDA approval in 2016.	[134,136,137]
**Tricyclo-DNA**	Preclinical—*mdx* mice	The efficacy of tricyclo-DNA was compared with PMO and 2OMePS in *mdx* mice (weekly IV injections for 12 weeks, up to 200 mg/kg/week): greater efficacy in exon skipping compared with the other treatments; restored dystrophin expression, particularly in the heart (up to 53%); improved respiratory function and skeletal muscle function.	[138]
**CPP-AOs-Pips**	Preclinical—*mdx* mice	Adult *mdx* mice were treated with different peptide-PMO (i.e., Pip5-PMO) conjugates, IM or IV administered: Pip5e-PMO was the most efficient in terms of exon 23 skipping (in skeletal muscle and heart); high dystrophin expression in skeletal muscle and particularly in the heart (>90%) after single IV injection (25 mg/kg). Repeated IV administrations of Pip6f-PMOs were delivered to *mdx* mice (10 mg/kg each dose): restored dystrophin in the skeletal muscles and heart and prevented exercise-induced cardiomyopathy.	[139,140]
**CPP-AOs-Phage Peptides**	Preclinical—*mdx* mice	Adult *mdx* mice were treated with SC injection of 2OMePS conjugated to 7-mer peptide (50 mg/kg, four times a week for 6 weeks): conjugation promoted uptake and exon skipping in skeletal muscles and heart.	[141]
**Possible other treatments and drug repositioning**			
**P188**	Preclinical—*mdx* CMs and mice	Copolymer poloxamer P188 was administered both in isolated dystrophic CMs from *mdx* mice and to *mdx* mice: reduction of stretch-induced calcium overload and increased compliance in vitro; improved cardiac haemodynamic performance in vivo also after dobutamine stress induction.	[142]
**MG53**	Preclinical—*mdx* mice	Recombinant human mitsugumin 53 (rhMG53) was injected IV and SC into adult *mdx* mice: reduced muscular pathology; prevention of exercise-induced damaged; no toxic effects.	[143]
**Sildenafil**	Preclinical—*mdx* mice. Clinical, Phase II—DBMD adults	Phosphodiesterase 5 inhibitor sildenafil was administered daily to *mdx* mice for 6 weeks: no alteration of cardiac haemodynamic parameters; decreased CM sarcolemmal injury (≈44%) after damage with isoproterenol. Sildenafil was daily administered for 14 weeks to *mdx* mice: increased diaphragm strength (≈15%) and anti-fibrotic effect; no impact on fatigue resistance; decreased muscular membrane permeability. In a clinical trial (NCT01168908), 20 adults MD patients were randomized to be treated with sildenafil (20 mg 3x daily) for 6 months, then additional 6 month treatment was administered to all patients: treated patients showed worsening of cardiac conditions (increased LVESV) and no statistically significan effect of the treatment was found; premature termination due to futility.	[144,145,146]
**Losartan**	Preclinical—*mdx* mice	Female *mdx* mice were treated with losartan for 6 months: decreased skeletal and cardiac muscle fibrosis; improved cardiac function (increased shortening fraction and reduced systolic blood pressure).	[147]
**Pirfenidone**	Preclinical—*mdx* mice	Aged *mdx* mice were treated with pirfenidone for 7 months: improved cardiac contractility and developed pressure and relaxation over time (±dP/dt); decreased level of TGF-β mRNA but no antifibrotic effect.	[148]
**Myostatin**	Clinical, Phase I/II—MD adults. Clinical, Phase II—DMD boys	Inhibition of myostatin/ActRIIB signaling pathway has been proven to improve dystrophic pathology in *mdx* mice and GRMD dogs preclinical models. In a clinical trial (NCT00104078), MYO-029, a neutralizing antibody for myostatin, was employed to treat 116 subjects with different types of MD; the compound was administered in different dosage (1, 3, 10 and 30 mg/kg) every two weeks for 6 months, followed by 3 months of follow up: assessed tolerability and safety; no improvement of muscle function. In another trial (NCT01239758) 11 DMD boys were treated with ACE-031 (0.5, 1.0 and 2.5 mg/kg) every 4 for 24 weeks: significant side effects were observed (e.g., nosebleeds, gum bleeding and dilated skin blood vessels); premature stop of the trial due to preliminary safety data.	[149,150]
**Urocortin**	Preclinical—*mdx* mice	Urocortins Ucn1 and Ucn2 were daily administered SC to young *mdx* mice for 2 weeks: improved muscle function; reduction of necrosis, particularly in the diaphragm; resistance to mechanical stress provoked by repetitive tetanization; contribution to calcium homeostasis.	[151]

IA, intra-arterial; IV, intravenous; IM, intramuscular; IP, intraperitoneal; IC, intramyocardial; LR, locoregional; SC, subcutaneous; CSA, total cross-sectional area; c.i., contraction induced; CM, cardiomyocyte; TA, tibialis anterior; ADM, abductor digiti minimi; MD, muscular dystrophy; LVESV, left ventricular end-systolic volume; 6MWD, 6-min-walk distance.
